# Supplementary material for: PLOS Computational Biology 2017 Reviewer and Editorial Board Thank You
Source: PLoS Comput Biol. 2018 Mar 15;14(3):e1006066. doi: 10.1371/journal.pcbi.1006066 (PMC5854228; doi:10.1371/journal.pcbi.1006066)
Supplement: S1 Editor List — (PDF) [file pcbi.1006066.s001.pdf]

PLOS | COMPUTATIONAL BIOLOGY

PLOS Computational Biology would like to thank all those who served on the Editorial Board in 2017:

Mark Alber
Samuel Alizon
Stefano Allesina
Russ B. Altman
Rustom Antia
Becca Asquith
Anand R. Asthagiri
Joseph Ayers
Joel Bader
Francesco P. Battaglia
Daniel A. Beard
Jeff Beck
Niko Beerenwinkel
Nir Ben-Tal
Matthias Bethge
Kim T. Blackwell
Gunnar Blohm
Sebastian Bonhoeffer
Elhanan Borenstein
Peer Bork
Rachel B. Brem
Steven E. Brenner
James M. Briggs
Daniel Bush
Shi-Jie Chen
Kevin Chen
K. Bretonnel Cohen
Aaron Darling
Jean Daunizeau
Rob J. De Boer
Bert L. de Groot
Charlotte M. Deane
Scott L. Diamond
Jörn Diedrichsen
Roland L. Dunbrack Jr.
Wolfgang Einhäuser
Jonathan A. Eisen
Michael B. Eisen
David Eisenberg
Arne Elofsson
Bard Ermentrout
Aldo A. Faisal
Matthew Ferrari
Jacquelyn S. Fetrow
Ernest Fraenkel
Christophe Fraser
James Gallo
Samuel J. Gershman
Lyle Graham
Helmut Grubmüller
Roderic Guigo
Boris S. Gutkin
Adrian M. Haith
Jason M. Haugh
Claus C. Hilgetag
Lilia M. Iakoucheva
Oleg A. Igoshin
Ilya Ioshikhes
Saad Jbabdi
Lars Juhl Jensen
Maricel G. Kann
Rachel Karchin
Peter M. Kasson
Alon Keinan
Ozlem Keskin
Roy Kishony
Dan Knights
Katia Koelle
Natalia L. Komarova
Konrad P. Kording
Sergei L. Kosakovsky Pond
Nikolaus Kriegeskorte
Morgan Langille
Peter E. Latham
Douglas A. Lauffenburger
Thomas Lengauer

Christina S. Leslie
Justin Lessler
Michael Levitt
Fran Lewitter
Jennifer Listgarten
James Lloyd-Smith
Jian Ma
Feilim Mac Gabhann
Jakob H. Macke
Alexander MacKerell
Philip K. Maini
Costas D. Maranas
Daniele Marinazzo
Scott Markel
Florian Markowetz
Alison L. Marsden
Marc A. Marti-Renom
Manja Marz
Frederick A. Matsen IV
John S. Mattick
Andrew D. McCulloch
Alice Carolyn McHardy
Martin Meier-Schellersheim
Daniel Mietchen
Satoru Miyano
Alexandre V. Morozov
Quaid Morris
Abigail Morrison
Qing Nie
Jens Nielsen
Kay Nieselt
William Stafford Noble
Ruth Nussinov
Yanay Ofran
Uwe Ohler
Jill O'Reilly
Christine A. Orengo
Francis Ouellette
Christos A. Ouzounis
Jason A. Papin
Mercedes Pascual
Mihaela Pertea
Bjoern Peters
Virginia E. Pitzer
Timothée Poisot
Andreas Prlic
Teresa M. Przytycka
Marco Punta
Predrag Radivojac
Christopher V. Rao
Benjamin J. Raphael
Jennifer L. Reed
Roland R. Regoes
Isidore Rigoutsos
Burkhard Rost
Andrey Rzhetsky
Marcel Salathé
Jeffrey J. Saucerman
Avner Schlessinger
Dina Schneidman
David B. Searls
Nicola Segata
Mona Singh
Donna K. Slonim
Joerg Stelling
Kai Tan
Mark M. Tanaka
Amos Tanay
Frédéric E. Theunissen
Denis Thieffry
Arne Traulsen
Olga Troyanskaya
Greg Tucker-Kellogg
David van der Spoel
Erik van Nimwegen
Cecile Viboud
Christian von Mering
Rebecca C. Wade
Edwin Wang
Guanghong Wei
Claus O. Wilke
Shoshana Wodak
Lingchong You
Weixiong Zhang
Sheng Zhong
Xianghong Jasmine Zhou
