# Supplementary material for: PLOS Computational Biology 2017 Reviewer and Editorial Board Thank You
Source: PLoS Comput Biol. 2018 Mar 15;14(3):e1006066. doi: 10.1371/journal.pcbi.1006066 (PMC5854228; doi:10.1371/journal.pcbi.1006066)
Supplement: S1 Guest Editor List — (PDF) [file pcbi.1006066.s002.pdf]

PLOS Computational Biology would like to thank all those who served as Guest Associate Editors in 2017:

Reka Albert
David Alonso
Patrick Aloy
Christian L. Althaus
Gary An
Alexander R. A. Anderson
Jean-Baptiste André
Jörn Anemüller
Behtash Babadi
Barbara M. Bakker
Ernest Barreto
Frederic Bartumeus
Danielle S. Bassett
Saonli Basu
Ulrik R. Beierholm
Christopher Benner
Matthew R. Bennett
Sven Bergmann
Carl T. Bergstrom
Max Berniker
Hugues Berry
Doron Betel
Vikas Bhandawat
Ran Blekhman
Silvia Blemker
Amitabha Bose
Dolores Bozovic
Jonathan R. Brennan
Yana Bromberg
Andre Brown
Nicolas Brunel
Javier Buceta
Harold A. Burgess
Daniel A. Butts
Patrick Cahan
Filippo Castiglione
Andrea Cavagna
Anushree Chatterjee
Marc Choisy
Stirling Churchman
Radoslaw Cichy
Caroline Colijn
Otto X. Cordero
Attila Csikász-Nagy
Hermann Cuntz
Eric N. Cytrynbaum
Daniel Damineli
Miles P. Davenport
Erik De Schutter
Andreas Deutsch
Xavier Didelot
Jonathan D. Dinman
Susanne Ditlevsen
Brent Doiron
Nikolay V. Dokholyan
Jean-Baptiste Durand
Eva Dyer
Leah Edelstein-Keshet
Gaute T. Einevoll
Udo A. Ernst
Ofer Feinerman
Brock Fenton
Neil M. Ferguson
Elana J. Fertig
Ione Fine
Jessica C. Flack
Sarel Fleishman
Jasmine Foo
Ashlee N. Ford Versypt
Paul Francois
Sebastian Funk
Alona Fyshe
Nicolas Galtier
Hector Garcia Martin
Karl Gegenfurtner
Mark B. Gerstein

Wulfram Gerstner
Dirk Gillespie
Anthony Gitter
Herve Glotin
Jeff Gore
Julian Gough
Roland Grafström
John M. Greally
Sonja Gruen
Ralf Haefner
Gerald Hahn
Turkan Haliloglu
Betz Halloran
Matthew Hartfield
Mitra J. Z. Hartmann
Moritz Helmstaedter
Matthias Helge Hennig
Matthias H. Hennig
Tomer Hertz
William Hlavacek
Thomas Höfer
Alexander Hoffmann
Haruo Hosoya
Lin Hou
C. Anthony Hunt
Bonnie L. Hurwitz
Trey Ideker
Cory Inman
Donald Jacobs
Joanna Jedrzejewski-Szmek
Ole Jensen
Robert L. Jernigan
Dezhe Z. Jin
Robert Johnston
Renaud Blaise Jolivet
Eric Jonas
Melissa L. Kemp
Anmar Khadra
Aly Khan
Steven H. Kleinstein
Andrew Knox
Jacob Koella
Roger Dimitri Kouyos
Gabriel Kreiman
Joachim Krug
Levin Kuhlmann
Hans-Peter Landolt
Christian Landry
Benjamin Langmead
Andrew J. Leigh Brown
Yaakov Koby Levy
Emmanuel Levy
Nathan E. Lewis
Marja-Leena Linne
Jin Liu
Nir London
Lit-Hsin Loo
Artur Luczak
David B. Lukatsky
William W. Lytton
Avi Ma'ayan
Radhakrishnan Mahadevan
Laurence T. Maloney
Pascal Mamassian
Adam H. Marblestone
Sergei Maslov
Thomas Matthews
John Mccarthy
Joe McIntyre
Julien Meaud
Ron Meir
Roeland M. H. Merks
Joshua N. Milstein
Stephen B. Montgomery
Jorrit Steven Montijn
Rani Moran
Viktor Müller
Chad L. Myers
Farzan Nadim
Felix Naef
Shikha Nangia
Peter Neri
Sander Nieuwenhuis
Pablo Padilla-Longoria
Bernhard O. Palsson
Anna R. R. Panchenko
Alexander V. Panfilov
Stefano Panzeri

Elena Papaleo
Il Memming Park
Arvind P. Pathak
Kiran Raosaheb Patil
Paul Pavlidis
Jian Peng
Alan S. Perelson
Thomas Pfeiffer
Adam M. Phillippy
Jonathan W. Pillow
Igor V. Pivkin
Joshua B. Plotkin
Nathan D. Price
Peng Qiu
Jose M. Ranz
Angela M. Reynolds
Arnd Roth
Yasser Roudi
Sushmita Roy
Julio Saez-Rodriguez
Francisco C. Santos
Herbert Sauro
Cristina Savin
Jeffrey Schank
Santiago Schnell
Paul Schrater
Ryan S. Senger
Reza Shadmehr
Vahid Shahrezaei
Eugene I. Shakhnovich
Jeffrey Shaman
Tomer Shlomi
Stanislav Shvartsman
Saurabh Sinha
Tobin Roy Sosnick
Rosangela Sozzani
David Sprinzak
Tanja Stadler
Carlos Stein
Dagmar Sternad
Volker Steuber
Ralf Steuer
Ian H. Stevenson
Kelly Suter
Chao Tang
Haixu Tang
Jing Tang
Jijun Tang
Pavel Tomancak
Marc Torrent
Zlatko Trajanoski
Todd W. Troyer
Krasimira Tsaneva-Atanasova
Tamir Tuller
Ten Tusscher
David Umulis
Minus van Baalen
Mark C. W. van Rossum
Paola Vera-Licona
Jean-Philippe Mr. Vert
Koen Vervaeke
Thomas Voets
Christine Vogel
Joshua Vogelstein
Vitaly Volpert
Anders Wallqvist
Harel Weinstein
Franz J. Weissing
Joshua S. Weitz
Benjamin Werner
Amy D. Willis
Dominik Wodarz
Daniel Krzysztof Wojcik
Catherine Wu
Zeba Wunderlich
Joao B. Xavier
Han Xu
Daniel Yamins
Tal Yarkoni
Esti Yeger-Lotem
Zhaolei Zhang
Bing Zhang
Xiang Zhou
Jun Zhu
