# Supplementary material for: PLOS Computational Biology 2017 Reviewer and Editorial Board Thank You
Source: PLoS Comput Biol. 2018 Mar 15;14(3):e1006066. doi: 10.1371/journal.pcbi.1006066 (PMC5854228; doi:10.1371/journal.pcbi.1006066)
Supplement: S1 Reviewer List — Alphabetical list of 2017 PLOS Computational Biology peer reviewers (A-D). (PDF) [file pcbi.1006066.s003.pdf]

PLOS Computational Biology would like to thank all those who reviewed on behalf of the journal in 2017:

Behnaam Aazhang
Ravinder Abrol
Luigi Acerbi
Guillaume Achaz
Giovanni Adami
Christoph Adami
Ben Adams
Miroslav Adzic
Ruedi Aebersold
Christof Aegerter
Jan Aerts
Ad Aertsen
Charles Affourtit
David Agard
Pratul Agarwal
Vikram Agarwal
Anurag Agrawal
Irma Aguilar-Delfin
Andrew Aguirre
Ehud Ahissar
Yashar Ahmadian
Alaa Ahmed
Xavier Aimé
James Aimone
Laurence Aitchison
Marco Ajelli
Thomas Akam
Schahram Akbarian
Sahar Akram
Tomas Alarcon
Larissa Albantakis
Frank Alber
Helen Alexander
Alexander Alexeev
Emil Alexov
Juan Alfonso Lopez
Lital Alfonta
Hamid Ali
Jun Allard
Carsten Allefeld
Genevera Allen
Colin Allen
Toby Allen
Eivind Almaas
Mohammad Al-Mamun
David Alonso
Patrick Aloy
Christian Althaus
Benjamin Althouse
Philipp Altrock
Stephen Altschul
Steven Altschuler
Yimy Amarillo
Yashar Amhadian
Gary An
Li An
Cheryl Andam
Alexander Anderson
Ingemar Andre
Massimo Andreatta
David Andrews
Steven Andrews
Thomas Andrillon
Ioannis Androulakis
Cécile Ané
Christof Angermueller
Danny Antaki
Péter Antal
Marco Antoniotti
Haroon Anwar
Stefano Anzellotti
Mikio Aoi
Miguel Aon
Juan Aparicio
Tomas Aragon
Alfonso Araque

Filippos Aravanopoulos
Roberto Araya
Hermenegild Arevalo
Markus Arnoldini
Jon Arnot
Gaurav Arya
Martin Aryee
Peter Arzberger
Hugues Aschard
David Ascher
Catherine Ascombe
Peter Ashcroft
Canan Atilgan
Teresa Attwood
Paul Atzberger
Elizabeth Au
Whitlow Au
Kin Fai Au
Rajeev Aurora
Robert Austin
Erin Austin
Andrea Avena-Koenigsberger
Bruno Averbeck
Jacob Axelsen
Ferhat Ay
Frank Aylward
Ettayapuram Ramaprasad Azhagiya Singam
Ramy Aziz
Baktash Babadi
Gabriele Babini
Joscha Bach
Rhonda Bacher
Nadine Bachmann
Jantien Backer
Albino Bacolla
Joel Bader
Gary Bader
Noemí Bahamontes-Rosa
Justin Bahl
Bahador Bahrami
Lu Bai
Marc Bailly-Bechet
Emily Baird
Travis Baker
Kevin Bakker
Stefan Balaz
Mahmoud Balbaa
William Balch
David Balduzzi
Oluwaseyi Balogun
Dan Bang
Martin Banks
Robert Banks
Vikas Bansal
Shweta Bansal
Pavel Baranov
Sergio Baranzini
Elisar Barbar
Nuno Barbosa-Morais
Alessandro Barducci
Izhar Bar-Gad
Debmalya Barh
Avner Bar-Hen
Somsubhra Barik
Sean Barnes
Caroline Baroukh
Andrea Barreiro
Douglas Barrick
Chaitanya Baru
Philippe Bastiaens
Eric Batchelor
Mark Bathe
Aaron Batista
Riza Batista-Navarro
Kayhan Batmanghelich
Demian Battaglia
Elena Battaglioli
Davide Bau
Chris Bauch
Amy Bauer
Victoria Bautch
Michael Baxa
Maxim Bazhenov
Jason Bazil
Robert Becker
Abbey Becker
Erin Becker
Travis Beckett

Christian Beckmann
Oliver Beckstein
Gerrit Beemster
Michael Beer
John Beggs
Marcelo Behar
Ulrik Beierholm
Tim Beissinger
George Bell
Pierre Bellec
Martine Ben Amar
Jan Benda
David Benkeser
Axel Benner
W. F. Drew Bennett
James Bennett
Daniel Bennett
Loisa Bennetto
Richard Benninger
Mikael Benson
Graeme Benstead-Hume
R. Bentley
B. Wayne Bequette
Philipp Berens
Florian Berger
Michael Berger
Christina Bergonzo
Alberto Bernacchia
Juliana Bernardes
Max Berniker
Hugues Berry
Richard Bertram
Olivier Bertrand
Robert Best
Andreas Beyer
Marianne Bezaire
Upinder Bhalla
Nita Bharti
Veer Bhatt
Rahul Bhui
Dapeng Bi
Dmitry Bibichkov
Martin Biehl
Maria Bighiu
Janine Bijsterbosch
James Binley
Kenneth Birnbaum
David Biron
Marc Birtwistle
Ilka Bischofs
Sebastian Bitzer
Guy Blanchard
Francois Blanquart
Michael Blinov
John Blischak
Jesse Bloom
Nils Blüthgen
Konstantin Blyuss
Rainer Böckmann
Rafal Bogacz
I. David Bogle
Peter Bolhuis
Erik Bollt
Luca Bolzoni
Maciej Boni
Richard Bonneau
Pascal Bonnet
Erie Boorman
Victoria Booth
Elhanan Borenstein
Christoph Borgers
Valentina Borghesani
Christine Borgman
Aladin Boriek
Alla Borisyuk
Benny Borremans
Alexander Borst
Kristofer Bouchard
Sebastien Bouret
Gregory Bowman
James Bown
Denis Boyer
Alan Boyle
Patrick Boyle
Patrick Bradley
Jan Brascamp
Robert Braun
Ignacio Bravo

Michelle Brazas
Romulus Breban
Nicolas Bredeche
Michael Breed
Rainer Breitling
Kevin Brennan
Eli Brenner
Helena Brentani
Assaf Breska
Gregory Brewer
Robert Brewster
Martin Brill
Tom Britton
Petter Brodin
Yana Bromberg
Logan Brooks
Andrew Brouwer
Andrea Brovelli
Sam Brown
James Brown
Brian Browning
Christoph Bruecker
Thijn Brummelkamp
Nicolas Brunel
Elizabeth Brunk
Bingni Brunton
Eleanor Brush
David Bryant
Vanni Bucci
Brad Buchsbaum
Michael Buck
Joshua Buckholtz
Ansgar Bueschges
Florian Buettner
Michael Buice
Edward Bullmore
Yoram Burak
Anthony Burkitt
Anat Burkovitz
Gordon Burleigh
Michael Busch
Harmen Bussemaker
Sarah Butcher
Daniel Butts
Helen Byrne
Danilo Bzdok
Juan Caballero-Pérez
Santiago Cadena
Gustavo Caetano-Anollés
Hayriye Cagnan
Patrick Cahan
Tunahan Cakir
Luz Irina Calderon Villalobos
Adam Calhoun
Vince Calhoun
Corrado Cali
Brian Camley
Stuart Campbell
Sharon Campbell
Carmen Canavier
Mark Cannell
Stefan Canzar
Yangxiaolu Cao
Yang Cao
Emidio Capriotti
Gabriel Cardona
Lucas Carey
Paolo Carloni
David Carlson
Martin Carpenter
Arkaitz Carracedo
Hannah Carter
Charles Carter Jr.
Francesco Paolo Casale
Jérome Casas
Gastone Castellani
Marco Catoni
Simon Cauchemez
Antonio Celani
Nihan Çelebi-Ölçüm
Michael Cerullo
Maurice Chacron
Benny Chain
Pinak Chakrabarti
John H. Challis
Ting-Fung Chan
Hue Chan
Chia-En Chang

Brad Chapman
Michael Chappell
Adam Charles
Vivek Charu
Steven Chase
Sonali Chaturvedi
Xiaoshu Chen
Steven Chen
Mengjie Chen
Zhuo Chen
Weitao Chen
Luonan Chen
Ken Chen
Chia-Chung Chen
Yeng-Long Chen
Bin Chen
Yong Cheng
Lu Cheng
Alan Cheng
Jianlin Cheng
Elizabeth Cherry
Allen Cheung
Vincent Cheung
Nicholas Chia
Dante Chialvo
Anush Chiappino-Pepe
Vikram Chib
George Chikenji
Lauren Childs
Shinung Ching
Rebecca Chisholm
Anton Chizhov
Dmitrii Chklovskii
Marc Choisy
Gaurav Chopra
Ching-Shan Chou
Carson Chow
Gerardo Chowell
Vassilios Christopoulos
Dominique Chu
Lon Chubiz
Thomas Churcher
Anne Churchland
Renata Ciereszko
Ali Cinar
Olivier Cinquin
Giovanni Ciriello
Luca Citi
Jean Clairambault
Nathan Clark
Timothy Clark
Benjamin Clark
Jason Climer
Claudia Clopath
Beth Clymer
Sarah Cobey
Simona Cocco
Robert Cockrell
Warren Code
Nick Cogan
Jay Coggan
Yann Cointepas
Steve Colburn
Todd Coleman
Caroline Colijn
Julio Collado-Vides
Giorgio Colombo
David Colquhoun
Lucy Colwell
Charles Connor
Jessica Conway
Alex Cook
Daniel Coombs
Jacques Corbeil
Otto X. Cordero
Manuel Corpas
Valentina Corradi
Francis Corson
Jesus Cortes
Carlo Cosentino
Ivan Costa
Rui Costa
James Costello
Justin Cotney
Michiel Cottaar
Antoine Coulon
Alexandre Courtiol
Peter Coveney

Lenore Cowen
David Cox
Katharine Coyte
Domenico Cozzetto
Patrick Crago
Keith Crandall
Frédéric Crevecoeur
Ivor Cribben
Armando Cuevas
Qinghua Cui
Aedín Culhane
Hermann Cuntz
Christina Curtis
Kit Curtius
Carina Curto
Marija Cvijovic
Eric Cytrynbaum
Tomer Czaczkes
Nuno da Costa
Onur Dagliyan
Harel Dahari
Kam Dahlquist
Vasilis Dakos
Alma Dal Co
Chiara Dalla Man
Janet Daly
Benjamin Dalziel
Egidio D'Angelo
Ramez Daniel
Noah Daniels
Bryan Daniels
Ludmila Danilova
Charles Danko
Phuong Dao
Arij Daou
Sayoni Das
Rhiju Das
Ranjan Dash
Xavier Daura
Justin Dauwels
Stephen David
William Davidson
Peter Davies
Yohan Davit
Ramana Davuluri
Nathaniel Daw
Troy Day
Peter Dayan
Lucilla de Arcangelis
Walter de Back
Rob De Boer
Alexandre De Brevern
Valerie de Crécy-Lagard
Vincent de Gardelle
Monique de Jager
Joao de Magalhaes
Nicola De Maio
Celso de Melo
Silvia De Monte
René De Mot
Saulo de Oliveira
Maurizio De Pittà
Gonzalo de Polavieja
Erik De Schutter
Marco De Vivo
Ksander de Winkel
Don DeAngelis
Eric Deeds
Nima Dehghani
Job Dekker
Paolo Del Giudice
Sara Del Valle
Guido Dell'Acqua
Simon Dellicour
Alex Demarsh
Thomas Deneux
Matthew Denwood
Mathieu Desroches
Christophe Dessimoz
Vincent Detours
Andreas Deutsch
Kushal Dey
Amir Dezfouli
Barbara Di Camillo
Antonella Di Pizio
Marco Di Stefano
Giovanni Di Veroli
Andreea Diaconescu

Ivy Dick
Frederic Dick
Jakob Dideriksen
Peter Diggle
Alexander Dimitrov
Jun Ding
Long Ding
David Dingli
Narendra Dixit
Peter Dodd
Charles Doering
Brent Doiron
Claudio Donati
Frank Dondelinger
Max Donelan
Tjibbe Donker
Christian Donner
Prashant Doshi
Zsuzsa Dosztányi
Nicholas Dotson
Carine Douarche
Kenji Doya
John Doyle
Andreas Dräger
Valentin Dragoi
Andrei Dragomir
Brian Drawert
David Drew
Guillaume Drion
William Drosopoulos
Shaul Druckmann
Jan Drugowitsch
D. Drummond
Julia Drylewicz
Fang Du
Yann Dufour
Denisa Duma
Michel Dumontier
Robin Dunbar
Mirek Dundr
Szonya Durant
Laurent Duret
Jonathan Dushoff
Simon Eaton
Tim Ebbels
Oliver Ebenhoeh
Blas Echebarria
Alexander Ecker
Leah Edelstein-Keshet
Matthew Edgington
Andrew Edwards
Felix Effenberger
Evelyn Eger
Charles Eggleton
Rosalind Eggo
S. Dusko Ehrlich
Anne Eichmann
Marisa Eisenberg
Adrian Elcock
James Elder
Coen Elemans
Mounya Elhilali
Julie Elie
Ardith El-Kareh
Jonathan Elkins
George Ellis
Kyle Ellrott
Emiley Eloe-Fadrosh
Arne Elofsson
Melissa Emery Thompson
David Enard
German Enciso
Mihaela Enculescu
Heiko Enderling
Denis-Alexander Engemann
Roger Enoka
Bastian Epp
Udo Ernst
Jason Ernst
Ananias Escalante
Umut Eser
Soheil Eshghi
Sebnem Essiz
Dario Estrin
Jocelyn Etienne
Mathew Evans
Arne Ewald
James Faeder

Luca Faes
Martin Falcke
Daniel Falush
Francesca Fanelli
Iman Farasat
José Faria
Farzad Farkhooi
Alessandro Farnè
Karoline Faust
Luis Favela
Evelina Fedorenko
Dmitry Fedosov
Christoph Feinauer
Adam Feist
David Fell
James Feng
Jake Ferguson
Andrew Ferguson
Fernando Fernandez
Aldo Ferrari
Alfredo Ferro
Mark Field
Christopher Fields
Ila Fiete
Hilary Finucane
Andrew Fiore-Gartland
Mark Fischer
Brian Fischer
Andras Fiser
Charles Fisher
David Fitzpatrick
Carlos Fitzsimons
Marc Fiume
Patrick Flaherty
Jennifer Flegg
Sarel Fleishman
Stephen Fleming
Roland Fleming
Ronan Fleming
Alexander Fletcher
Patrick Fletcher
Vincent Fleury
Juan Florez Weidinger
Anthony Fodor
Aaron Fogelson
Antonio Pedro Fonseca
Jasmine Foo
Daniel Forger
Elia Formisano
Pau Formosa-Jordan
Arianna Fornili
Alex Fornito
Nikolaus Fortelny
Guillaume Fournié
Christina Fournier
Alessio Franci
Michael Frank
Daniel Franks
Nigel Franks
Gareth Fraser
Erin Fraser
Franca Fraternali
Jeffrey Fredberg
Luke Fredette
Thomas Freeman
Dean Freestone
Winrich Freiwald
Erwin Frey
Iddo Friedberg
Jonathan Friedman
Erwin Frise
Karl Friston
Chris Frith
Martin Frith
Helmchen Fritjof
Fabian Fröhlich
Holger Fröhlich
H. Frost
Simon Frost
Feng Fu
Ayari Fuentes-Hernandez
Koichi Fujimoto
Masaya Fujita
Keiichi Fukaya
Atsushi Fukushima
Ben Fulcher
Isaac Chun-Hai Fung
Sebastian Funk

Nicholas Furnham
Norbert Furtmann
Diana Fusco
Toni Gabaldon
Ryan Gabrys
Krzysztof Gajowniczek
Roberto Galán
Joseph Galea
Clovis Galiez
Juan Álvaro Gallego
Pedro Gallego
Emilio Gallicchio
Oxana Galzitskaya
Gowrishankar Ganesh
Vitaly Ganusov
Chunfang Gao
Victor Garcia
Miguel Garcia-Cabezas
Krishna Garikipati
Romain Garnier
Simon Garnier
Ruben Garrido Oter
Eleftherios Garyfallidis
Olivier Gascuel
Pascale Gaudet
Jeffry P. Gavornik
Hao Ge
Philipp Gebhardt
Nils Gehlenborg
Lendert Gelens
Gambardella Gennaro
Andrew Gentles
Lisa Genzel
Odile Gerard
Philip Gerlee
Philip Gerrish
Mark Gerstein
Jana Gevertz
David Gfeller
Mohamed Ghadie
Maarten Ghequire
Gavin Gibson
Martin Giese
Giorgio Gilestro
Peter Gillespie
Val Gillet
Jesse Gillis
Matthieu Gilson
Francesco Ginelli
Andrea Giometto
Andrea Giovannucci
Benoît Girard
Andreas Gisel
Simon Giszter
Rosalba Giugno
Alessandro Giuliani
Julijana Gjorgjieva
Jan Gläscher
Leon Glass
Ingmar Glauche
Gustavo Glusman
Peter Gmeiner
Alberto Gobbi
Keith Godfrey
Adam Godzik
Pranay Goel
Lea Goentoro
Yana Gofman
Joshua Goldberg
Anna Goldenberg
Mark Goldman
Joshua Goldwyn
Tim Gollisch
Leonardo Gollo
David Golomb
Gabriela Gomes
Shawn Gomez
Jorge Gomez Tejeda Zanudo
Alex Gomez-Marin
Gerhard Gompper
Benjamin Good
David Goode
Chandrasekhar Gopala
Jeff Gore
Andrew Goryachev
Katie Gostic
Assaf Gottlieb
Fred Gould

Nir Gov
Carlos Gracia-Lazaro
Trevor Graham
Bruce Graham
Alexandre Gramfort
Francois Graner
Gianvito Grasso
Michael Graupner
Simon Gravel
Frederik Graw
Alexander Green
Jeremy Green
David Greenberg
Michelle Greene
Bryan Greenhouse
Joseph Greenstein
Celia Greenwood
Megan Greischar
Anna Greka
Thomas Griffiths
Andrey Grigoriev
Irina Grigorova
Jacopo Grilli
Ramon Grima
Mark Grimes
Adam Gristwood
Anna Grosberg
Alan Grossfield
Christina Grozinger
Jelena Grujic
Yongtao Guan
Steinn Gudmundsson
Jeremie Guedj
Justin Guinney
Caterina Guiot
Tanuj Gulati
Alexander Gultyaev
James Gumbart
Rudiyanto Gunawan
Emre Guney
Kris Gunsalus
Ulrich Günther
Daqing Guo
Feng-Biao Guo
Surya Gupta
Alexander Gusev
Robert Gütig
Miklos Guttman
Olgun Guvench
Robert Guy
Melissa Gymrek
Sandor Gyorke
Joon Ha
Julie Haas
Michael Habeck
Noushin Hadadi
Lilach Hadany
Ralf Haefner
Maximilan Haeussler
Stephen Hagen
Juergen Hahn
Benjamin Haibe-Kains
Vincent Hakim
Tariq Halasa
Matthew Hall
Bjarni Halldórsson
Mark Hallen
Jeanette Hällgren-Kotaleski
Geir Halnes
Marc Halushka
Liberty Hamilton
Fred Hamker
Fred Hamprecht
Buhm Han
Andreas Handel
Sridhar Hannenhalli
Thomas Hansen
Ulrich Hansmann
Bryan Hanson
Wenrui Hao
Murali Haran
William Harcombe
Todd Hare
Michael Harms
Kenneth Harris
Todd Harris
Andrew Harris
Traver Hart

Joachim Hass
Haralampos Hatzikirou
Bernard Haubold
Jason Haugh
Stefan Häusler
Robin Haw
Rhoda Hawkins
Andrea Hawkins-Daarud
Satoru Hayasaka
Ben Hayden
Heather Hayenga
Molly Hayes
Jennifer Hays
Steven Hayward
Qixin He
Zihuai He
Zengyou He
Simon Heath
Martin Hebart
Laurent Hébert-Dufresne
Charles Heckman
Ellie Heckscher
Dieter Heermann
Dominik Heider
Jordi Heijman
Eva Heinz
Rebecca Heise
Laura Heiser
Leonhard Held
Moritz Helias
Stefan Hellander
Peter Hellyer
Gibran Hemani
Adam Hendricks
Ron Henkel
Christian Henneberger
Guillaume Hennequin
James Hennessy
Matthias Hennig
Christopher Henry
Joshua Herbeck
James Herbert-Read
Esteban Hernandez-Vargas
Malte Herold
Markus Herrgard
John Hertz
Andreas Herz
David Herzfeld
Franz Herzog
Kathryn Hess
James Hetherington
Steven Hill
Alison Hill
Benjamin Hillmann
Tsuyoshi Hirota
Gordon Ho
Yen-Yi Ho
Oliver Hobert
Gabriel Hoffman
Alexander Hoffmann
Jan-Hendrik Hofmeyr
Christopher Hogue
David Holcman
Christopher Holdgraf
Liisa Holm
William Holmes
Susan Holmes
Knut Holthoff
Hermann-Georg Holzhütter
Hisao Honda
Christian Hong
Ilka Hoof
David Hoppe
Aidan Horner
Amnon Horovitz
Peter Horvath
Chen Hou
Xueliang Hou
John Houde
Eivind Hovig
Clare Howarth
Nathanael Hoze
Hua Hu
Yiming Hu
Ming Hu
Christopher Huang
Kerwyn Huang
Xuhui Huang

Haiping Huang
Chris Huang
Bin Huang
Jochen Hub
Robert Hubley
D. L. Hudson
Lars Hufnagel
Brent Hughes
Mark Humphries
Cendri Hutcherson
Axel Hutt
Wonmuk Hwang
Changbong Hyeon
Aapo Hyvarinen
Giandomenico Iannetti
Susan Idicula-Thomas
Christopher Illingworth
Petra Imhof
Robin Ince
Giles Innocent
Michael Inouye
Christos Ioannou
Ed Ionides
Francesco Iorio
Jaime Iranzo
Manish Issar
Laura Itzhaki
Shalev Itzkovitz
Renata Ivanek
Plamen Ivanov
Jun Izawa
Sophie Jackson
Robert Jacobs
Anders Jacobsen
Mohsin Jafri
Neema Jamshidi
Kevin Janes
Sarath Chandra Janga
Julian Jara-Ettinger
Alfonso Jaramillo
Peter Jedlicka
Paul Jensen
Oliver Jensen
Robert Jernigan
Antoine Jerusalem
Changge Ji
Gengjie Jia
Yuchao Jiang
Yi Jiang
Hui Jiang
Peng Jiang
Yuling Jiao
Anupama Jigisha
Alexandra Jilkine
Dezhe Jin
Victor Jin
Viktor Jirsa
Michael Johansson
Daniel Johnson
Gregory Johnson
Heath Johnson
Wilsaan Joiner
Vladimir Jojic
Mario Jolicoeur
Renaud Jolivet
Clare Jolly
Liz Jones
Stephanie Jones
C. Victor Jongeneel
Maxwell Joseph
Badal Joshi
Kresimir Josic
Krešimir Josić
Marko Jovanovic
Richard Judson
Timothy Julian
Igor Jurisica
Shabnam Kadir
Thorsten Kahnt
Kozo Kaibuchi
Marcus Kaiser
Shubha Kale
Stiliyan Kalitzin
Kaiwen Kam
Colince Kamdem
Sasikiran Kandula
Hyun Min Kang
Natarajan Kannan

Rowland Kao
David Kappel
Ezgi Karaca
Esin Karahan
Peter Karp
Jonathan Karr
Istvan Karsai
Ilene Karsch-Mizrachi
Matthias Kaschube
Mamoru Kato
Kamran Kaveh
Mitsuo Kawato
Ruian Ke
Robert Kearney
Stephen Keeley
David Kelley
Chris Kello
Janet Kelso
Carsten Kemena
Melissa Kemp
Christopher Kempes
Ephraim Kenigsberg
David Kennedy
Mehdi Keramati
Eduard Kerkhoven
Paul Kersey
Anisha Keshavan
Can Kesmir
Ioannis Kevrekidis
Anmar Khadra
Mehdi Khamassi
Mustafa Khammash
Peter Kharchenko
Firas Khatib
Hossein Khiabanian
Kamran Khodakhah
David Khoury
Natalia Khuri
Akinori Kidera
Stefan Kiebel
Tim Kietzmann
Daisuke Kihara
Takeshi Kikuchi
Alastair Kilpatrick
A. Kilpatrick
Kyoung Kim
Jaebum Kim
Minsu Kim
Jin-Dong Kim
Dokyoon Kim
Jangwon Kim
Philip Kim
Taeyoon Kim
Yangjin Kim
Molly King
Malcolm King
David Kirchman
Natalia Kirienko
Vivien Kirk
Paul Kirk
Denise Kirschner
Steffen Klamt
Eric Klann
Isaac Klapper
Gunnar Klau
Jeffery Klauda
Eili Klein
David Kleinfeld
Miriam Klein-Flügge
Jens Kleinjung
Vaclav Klika
Peter Klimek
Bertram Klinger
Barbara Klink
David Klinke
Edda Klipp
Edda Kloppmann
Achim Klug
Stefan Klumpp
Sebastian Kmiecik
David Knowles
Andrew Knox
Frank Koch
Juergen Koefinger
Hiroyuki Koga
Hiroshi Kohsaka
Ai Koizumi
Peter Kok

Sepp Kollmorgen
Rachel Kolodny
Anton Komar
Arun Konagurthu
Hidetoshi Kono
Simon Koplev
Jan Korbel
Dmitry Korkin
Alon Korngreen
Katja Kornysheva
Kirill Korolev
Steven Kosak
David Koslicki
Mickey Kosloff
Lubomir Kostal
Raphael Koster
Roger Kouyos
Jovana Kovacevic
Gregor Kovacic
Michael Koval
Mehmet Koyuturk
Dima Kozakov
Ian Krajbich
Frederick J. Krambeck
Mark Kramer
Pamela Kreeger
Mirjam Kretzschmar
Jeffrey Krichmar
Giri Krishnan
Arjun Krishnan
Timm Krueger
Meghana Kshirsagar
Rui Kuang
Jonas Kubilius
James Kubricht
Adam Kucharski
Grzegorz Kudla
Rolf Kuemmerli
Leslie Kuhn
Michael Kuhn
Denise Kühnert
Pavel Kuksa
Rahul Kulkarni
Arvind Kumar
Manish Kumar
Anuj Kumar
Anne Kupczok
Yasutaka Kurata
Wataru Kurebayashi
Katharina Kusejko
Kwame Kutten
Christina Kuttler
Alexey Kuznetsov
Giancarlo La Camera
Alain Laederach
Oliver Laeyendecker
Kevin Lafferty
Megan Lafollette
Leo Lahti
Samuel Lai
Guillaume Lambert
Ganhui Lan
David Landsman
Michael Landy
Angela Langdon
Peter Langfelder
Georg Langs
Markus Lappe
Matthew Larkum
Camilla Larsen
Ola Larsson
Kenneth Latimer
Ken Lau
Richard Lavery
Jeffrey Lawrence
Matthew Lazzara
Rodney Lea
Camille Lebarbenchon
Sang Wan Lee
Pilhwa Lee
Uncheol Lee
Su-In Lee
Michael Lee
Jooyoung Lee
Philippe Lefèvre
Laurent Lehmann
Jeremy Leipzig
Mark Leiserson

Thomas Leitner
Christopher Lemmon
Mark Lemmon
Máté Lengyel
Boris Lenhard
Suzanne Lenhart
Bart Lenselink
David Lentink
Marilisa Leone
Nathan Lepora
Bluma Lesch
Mark Lescroart
Till Lesker
Erez Levanon
Andre Levchenko
Gabriel Leventhal
Anna Levina
Michael Levine
Herbert Levine
Emmanuel Levy
Ron Levy
Yaakov Levy
Oded Lewinson
Nathan Lewis
Jarrod Lewis-Peacock
Joel Lexchin
Zhengwei Li
Guoshi Li
Yong Li
Yanan Li
Shitao Li
Ho Ling Li
Min Li
Yue Li
Yun Li
Heng Li
Hu Li
Jie Liang
Chung-Shou Liao
Ted Liefeld
Sukbin Lim
Xubo Lin
Scott Linderman
Jennifer Linderman
Rune Linding
Kresten Lindorff-Larsen
Alexandre Linhares
Marja-Leena Linne
Tal Linzen
Sébastien Lion
Jan Lipfert
Jan Liphardt
Tomasz Lipniacki
Christoph Lippert
Hans Joachim Lipps
Ashok Litwin-Kumar
Jian Liu
Chuanhai Liu
Hong Liu
Xinfeng Liu
Tao Liu
Yang-Yu Liu
Ke Liu
Hesheng Liu
Qi Liu
Hao Liu
Joseph Lizier
Manuel Llinas
David Lloyd
Eric Lock
Axel Loewe
Eric Lofgren
Po-Ru Loh
Georgios Lolas
Eric Londin
Nir London
Quan Long
Manuel Lopes
Fernando Lopes da Silva
Carlos Lopez
Evangelina López de Maturana
Ronny Lorenz
Tommaso Lorenzi
Matthieu Louis
Eugen Lounkine
Yoram Louzoun
Michael Love
Claude Loverdo

Craig Lowe
John Lowengrub
Ting Lu
Jiahai Lu
Long Lu
Zhiyong Lu
Mingyang Lu
Sharon Lubkin
Peter Lucas
Fabio Luciani
Georg Luebeck
Ben Luisi
Aaron Lun
Claudia Lunghi
Cheng Ly
Samantha Lycett
George Lykotrafitis
Ao Ma
Avi Ma'ayan
Ben MacArthur
Ruth Mace
Harold MacGillavry
Christian Machens
Steven Mack
Paul Macklin
Adam MacLean
Tom Madden
Anotida Madzvamuse
Carsten Magnus
Krisztian Magori
Finlay Maguire
Radhakrishnan Mahadevan
Swapnil Mahajan
Tokameh Mahmoudi
Francois Major
Michael Mak
Madhav Mani
Jeremy Manning
Michael Manookin
Kamel Mansouri
Massimo Mantegazza
Jessica Mar
Madhav Marathe
Daniel Marbach
Jonathan Marchini
Anna Marciniak-Czochra
Eve Marder
Adil Mardinoglu
Daniel Margulies
Mahendra Mariadassou
R. Mariano
Michael Marks
Fritz Markwardt
Phillip Marlow
Olivier Marre
Joseph Marsh
Donald Marsh
James Marshall
Pier Luigi Martelli
Andrea Martin
Micaela Martinez
Pamela Martinez
Alfonso Martinez-Arias
Encarnacion Martinez-Salas
Georg Martius
Sasai Masaki
Sergei Maslov
Timothée Masquelier
Marcello Massimini
Paolo Massobrio
Naoki Masuda
Matthew Matell
Alexander Mathis
Christoph Mathys
Vera Matser
Tomoaki Matsuura
Maurizio Mattia
Victor Matveev
Tatiana Maximova
Abigail Mayes
Heather Mayes
Arya Mazumdar
Alberto Mazzoni
Mark McAuley
James McCaw
Helen McCreery
Andrew McDavid
Donald McElwain

Johanna McEntyre
Christopher McFarland
Sarah McGough
Nicholas McGranahan
Myles McLaughlin
Richard McNally
Daniel McNamee
Fiona McRobb
Graham McVicker
Jason Mears
Rebecca Mease
Julien Meaud
Milagros Medina
Sean Megason
Wout Megchelenbrink
Murad Megjhani
Pankaj Mehta
Ron Meir
Christian Meisel
Jorge Mejias
Carlos Melian
Jack Mellor
Joerg Menche
Pedro Mendes
Marc Mendillo
Sebastián Mendoza
Jorge Menendez
Gautam Menon
Vilas Menon
Josh Merel
Roeland Merks
Stefano Merler
Robert Merrison
Martha Merrow
Frederic Mery
Jamel Meslamani
Arnaud Messé
Ann Meyer
Sebastian Meyer
Axel Meyer
Thomas Meyer
Martin Meyer
Michelle Meyer
Pieter Meysman
Huaiyu Mi
Chris Miall
Matthew Michalska-Smith
Audrey Michel
Stephen Michnick
Richard (Rick) Michod
Tom Michoel
Sarah Middleton
Dale Mierke
Michele Migliore
Alexey Mikaberidze
Tijana Milenkovic
Andreas Milias-Argeitis
Paul Miller
Christopher Miller
Samuel Minot
Konstantin Mischaikow
Anusha Mishra
Bratislav Misic
Hitesh Mistry
Grant Mitchell
Maria Miteva
Sorin Mitran
Sara Mitri
Antonina Mitrofanova
Jeetain Mittal
John Mittler
Babak Momeni
Juliet Moncaster
Gianluigi Mongillo
Jonathan Monk
Daniel Montoya
Alana Moore
J. Bernadette Moore
Thierry Mora
Serge Morand
Ion Moraru
Faruck Morcos
Ernesto Mordecki
Ryan Morehead
Ruben Moreno-Bote
Miguel Moreno-Risueno
Paolo Moretti
Yoichiro Mori

Stefano Morotti
Adam Morris
Garrett Morris
Kendall Morris
David Morrison
Roberto Mosca
Philip Mosier
Mathieu Moslonka-Lefebvre
Thiago Mosqueiro
Sara Mostafavi
Sarah Mostafavi
Gholam Motamedi
Allan Motyer
Carina Mugal
Andrew Mugler
Eran Mukamel
Nicola Mulder
Jeanette Mumford
Christopher Mungall
Monica Munoz-Torres
Paul Munro
Brian Munsky
T. Murali
John Murray
Ben Murrell
Arvind Murugan
David Muscatello
Cameron Musco
Ramaiah Muthyala
Akira Muto
Ekaterina Myasnikova
Nicholas Myers
Christopher Myers
Iftach Nachman
Suhita Nadkarni
Kristen Naegle
Srikantan Nagarajan
Rana Nagarkatti
Marcos Nahmad
Laleh Najafizadeh
Haruki Nakamura
Vikas Nanda
Manjari Narayan
Rishikesh Narayanan
Matthew Nassar
Samuel Nastase
Richard Naud
Grzegorz Nawrocki
Martin Nawrot
Chris Neale
Henry Neeman
Keith Neeves
Richard Neher
Ilya Nemenman
Adrian Nestor
Gregor Neuert
Samuel Neymotin
Michael Ng
Steven Niederer
Morten Nielsen
Dylan Nielson
Noushin Niknafs
Michael Nikolaou
Zoran Nikoloski
Lennart Nilsson
Mahesan Niranjan
Ilana Nisky
Yael Niv
Robert Noble
William Noble
Christopher Nogiec
Stefano Nolfi
Elad Noor
Imran Noorani
Uta Noppeney
Jared Nordman
Yohei Norimatsu
Sergei Noskov
Thomas Nowotny
Andrew Noymer
Daichi Nozaki
Sergey Nurk
Ruth Nussinov
Duane Nykamp
Daibhid O Maoileidigh
Diarmuid Ó'Maoiléidigh
Mike Oaksford
Marcel Oberlaender

Jonas Obleser
Dominik Obrist
Sean O'Callahan
Brian O'Connor
David Odde
Timothy O'Donnell
Cian O'Donnell
James O'Dwyer
Dietmar Oelz
Layla Oesper
Dimitri Ognibene
Masafumi Oizumi
Blessing Ojeme
Yasumasa Okada
Michael Okun
Freyja Olafsdottir
Williams Olbricht
Umberto Olcese
Timothy O'Leary
Guilherme Oliveira
Noah Olsman
Elizabeth Olson
Matthew O'Meara
Jose Onuchic
Alexey Onufriev
Lulla Opatowski
Madeleine Opitz
Jean-Jacques Orban De Xivry
Hatice Osmanbeyoglu
Srdjan Ostojic
Niels Otani
Hans Othmer
Melanie Ott
Ross Otto
Rachid Ounit
Diego Oyarzún
Banu Ozkan
Stephan Pabinger
Marius Pachitariu
Emanuele Paci
Ranjith Padinhateeri
Katrin Paeschke
Andrea Pagnani
Hui-Nam Pak
Gianmarco Palamara
Margriet Palm
Stephanie Palmer
Stefano Palminteri
Matias Palva
Chenyi Pan
Anna R. Panchenko
Aridaman Pandit
Xiaodong Pang
Sergio Pantano
Robert Pantazes
Casian Pantea
Mikhail Panteleev
Stefano Panzeri
Jason Papin
Athanasia Papoutsi
Jun Won Park
Il Park
Ulrich Parlitz
C. Alejandro Parraga
Leopold Parts
Claude Pasquier
Alexander Pastukhov
Anitha Pasupathy
Ankit Patel
Benedict Paten
Pras Pathmanathan
Kiran Patil
Pintu Patra
Rob Patro
Jonas Paulsen
Walter Paulus
Pornntiwa Pawara
Joshua Payne
William Pearson
Dana Pe'er
Bradford Peercy
Lucia Peixoto
Lorenzo Pellis
Jorge Peña
Rafael Pena-Miller
Jian Peng
Zhangli Peng
Hanchuan Peng

William Penny
Kim Pepin
Martin Pera
Matjaz Perc
Herbert Peremans
Alberto Perez
Alfonso Perez Alfonso
Ruben Perez-Carrasco
Víctor Pérez-García
F. Pérez-Rodríguez
Juan Perilla
Vipul Periwal
Theodore Perkins
Alex Perkins
Fernando Peruani
Graziano Pesole
Brenden Petersen
Spase Petkoski
Adrien Peyrache
Remi Peyraud
Nico Pfeifer
Benjamin Pfeuty
Jean-Pascal Pfister
Steven Phillips
Stefano Piana
Eugenio Piasini
Florian Pieper
Arkady Pikovsky
Satish Pillai
Jonathan Pillow
Praveen Pilly
Luca Pinello
Dimitris Pinotsis
Davide Piovesan
Jörn-Steffen Pischke
Angela Oliveira Pisco
Zygmunt Pizlo
Cinzia Pizzi
Francisco Planes
Dietmar Plenz
Eftychios Pnevmatikakis
Frank Poelwijk
Michael Poidinger
Jan Poleszczuk
Chiara Poletto
Anastasia Politou
Thomas D. Pollard
Katherine Pollard
David Pollock
Régis Pomès
Julia Ponomarenko
Yann Ponty
Hoifung Poon
Art Poon
Bernd Porr
Alexandre Pouget
Christophe Pouzat
Juan Poyatos
Shyam Prabhakar
Judy Prasad
John Preisser
L. Premo
Alexis Prevost
Nathan Price
David Price
Morgan Price
Nicholas Price
Viola Priesemann
Sylvain Prigent
Boris Prilutsky
Yuri Pritykin
U. Deva Priyakumar
Andreas Prlic
Stephen Proulx
Nicholas Provart
Emily Prud'hommeaux
Kay Prüfer
Sunil Puria
Xiaoning Qian
Yan Qin
Zhaohui Qin
Peng Qiu
Zhilin Qu
Francisco Quintana
Gerald Quon
Eva Qwarnstrom
Diego R. Barneche
Richard Rabbitt

Ravi Radhakrishnan
Tomas Radivoyevitch
Anu Raghunathan
Manon Ragonnet-Cronin
Rayees Rahman
Sven Rahmann
Indika Rajapakse
Nasir Rajpoot
Joseph Ramsey
Avinash Ramu
Amanda Randles
Sushil Rane
Sridhar Ranganathan
Nicolas Rascovan
Parisa Rashidi
David Rasmussen
Fraydoon Rastinejad
Oliver Ratmann
Frank Rattay
Andreas Raue
Josef Rauschecker
Benedikt Rauscher
Sarah Rauscher
Debashree Ray
Evan Ray
Brandon Razooky
Mark Read
Elizabeth Read
Mario Recker
A. David Redish
Floyd Reed
Louis Reese
Adam Reid
Robert Reiner
Knut Reinert
Katarzyna Rejniak
Angela Relógio
Justin Remais
Michiel Remme
Jian Ren
Bernhard Renard
Osbaldo Resendis-Antonio
Yakir Reshef
Juan Restrepo
Boris Reva
Alex Reyes
Jean-Louis Reymond
Andrew Reynolds
Ruy Ribeiro
Blake Richards
Gerard Ridgway
Hermann Riecke
Mattia Rigotti
Lionel Rigoux
Todd Riley
Steven Riley
John Rinzel
Daniel Ripoll
Herre Jelger Risselada
Olivia Rissland
Matthew Ritchie
Petra Ritter
Daniel Riveline
Elijah Roberts
David Robertson
Marc Robinson-Rechavi
Paul Robustelli
Miguel Rocha
Mathieu Roche
Benjamin Roche
Kathy Rockland
Russell Rockne
Guillermo Rodrigo
Joao Rodrigues
Ricardo Rodríguez de la Vega
Ignacio Rodriguez-Brenes
Raul Rodriguez-Esteban
Adrienne Roeder
Tomasz Rog
Simon Rogers
Igor Rogozin
Remo Rohs
Johann Rohwer
Pawel Romanczuk
Sandro Romani
Monica Romero Lopez
Rafael Romero-Garcia
Libin Rong

Marianne Rooman
Glen Ropella
Marcus Roper
Mary Rorick
Robert Rosenbaum
Joshua Ross
Edith Ross
Christian Rössert
Edina Rosta
Daniel Rotroff
Horacio Rotstein
Stefan Rotter
Elsa Rousseau
Marc Roussel
Igor Rouzine
Alex Roxin
Amitava Roy
Sushmita Roy
Christopher Rozell
Leonid Rubchinsky
Florian Rubelt
Frank Rudzicz
Elmar Rueckert
Sten Ruediger
Matthew Ruffalo
Sandra Rugonyi
Marilyn Ruiz
Nick Ruktanonchai
Peter Ruoff
C. Rupar
Eytan Ruppin
Evan Russek
Charles Russell
Colm Ryan
Robert Ryan
Ilya Rybak
Pedro Saa
David Saakian
Hannes Saal
Yuri Saalmann
Francois Sabot
Nasim Sabouri
Maria Pia Saccomani
João Sacramento
Anguraj Sadanandam
Farrah Sadre-Marandi
Julio Saez-Rodriguez
Dov Sagi
Surya Saha
Özgür Sahin
Supreet Saini
Lucas Salas
Luisa Salazar
Andrej Sali
Henrik Salje
Leena Salmela
Nathan Salomonis
Julia Salzman
Md. Abul Hassan Samee
Kazuyuki Samejima
Deanne Sammond
Somponnat Sampattavanich
Natalia Sanchez
Fatima Sanchez-Cabo
Thomas Sandercock
Jean-Christophe Sandoz
Sanjay Sane
Fidel Santamaria
Mauricio Santillana
Fernando Santos
Mihaela Sardiu
Masaki Sasai
Rahul Satija
Leslie Satin
Kei Sato
Herbert Sauro
Thomas Sauter
Richard Savage
Yonatan Savir
Stephen Sawiak
Samuel Scarpino
Manuel Schabus
William Schafer
Jeffrey Schall
Anna Schapiro
Hansjörg Scherberger
Nico Scherf
Richard Scheuermann

Marc Schieber
Helmut Schiessel
William Schiesser
Steven Schiff
Marcel Schilling
Tamar Schlick
Alexander Schliep
Jonas Schluter
Katharina Schmack
Andre Schmidt
Joern Schmiedel
Vicky Schneider
Reinhard Schneider
Dina Schneidman
Santiago Schnell
David Schoenfeld
Robert Schöpflin
Manuel Schottdorf
Joshua Schraiber
Paul Schrater
Gideon Schreiber
Alison Schroer
Johannes Schropp
Jannis Schuecker
Stephan Schürer
Heiko Schütt
Frederic Schutz
Tilo Schwalger
Russell Schwartz
Gregory Schwartz
Roland Schwarz
Cornelius Schwarz
Nicolas Schweighofer
Ulrich Schweizer
Patric Schyman
Joost Schymkowitz
Pat Scott
Jacob Scott
Paola Sebastiani
Timothy Secomb
Luigi Sedda
Idan Segev
Eyal Seidemann
Aaron Seitz
Allen Selverston
Ryan Senger
Durba Sengupta
Cathal Seoighe
Chaok Seok
Oliver Serang
Thomas Serre
Sohan Seth
Stefano Severi
Nikolaos Sgourakis
Ioannis Sgouralis
Dhaval Shah
Babak Shahbaba
Maoz Shamir
Karthik Shankar
B. Jesse Shapiro
Maxim Shapovalov
Anukriti Sharma
Amit Sharma
Vivek Sharma
Tali Sharot
Kim Sharp
Tatyana Sharpee
Megan Shaw
Sidney Shaw
Alexey Shaytan
David Sheinberg
Arthur Sherman
Woodrow Shew
Lei Shi
Kazuhisa Shibata
Denis Shields
Heejung Shim
Hideaki Shimazaki
James Shine
Shigeru Shinomoto
Patrick Shipman
Suzanne Shoffner
Sourya Shrestha
Indira Shrivastava
Hari Shroff
Joshua Shrout
Blerta Shtylla
George Shubeita

Stanislav Shvartsman
Kyriaki Sidiropoulou
Hartwig Roman Siebner
Ricardo Silva
R. Silver
Justin Silverman
Patrick Simen
Jonathan Simon
Beth Simon
Eero Simoncelli
Thomas Simonson
Matthew Simpson
Saurabh Sinha
Robert Sinsabaugh
Albert Siryaporn
Malcolm Slaney
Hamutal Slovin
Joanna Slusky
Bruce Smaill
Neil Smalheiser
Colin Smith
Jeffrey Smith
David Smith
Thomas Smith
Gordon Smyth
Kim Sneppen
James Sneyd
Jacky Snoep
Alexander Sodt
Johannes Soeding
Christian Soeller
Peter Sokol-Hessner
Claudia Solis-Lemus
Trygve Solstad
Haim Sompolinsky
Francis Song
Chi Song
Nikolaus Sonnenschein
Amika Sood
Fabian Soto
Samuel Soubeyrand
Orkun Soyer
Doug Speed
Nora Speicher
Sabrina Spencer
Ian Spicknall
Stephanie Spielman
Tommy Sprague
Henning Sprekeler
Kevin Staras
Rory Stark
Andrzej Stasiak
Melanie Stefan
Johannes Stegmaier
Gary Stein
Martin Stemmler
Angelique Stephanou
John Stevens
Ian Stevenson
Alexander Stewart
Mark Steyvers
Jonathan Stieglitz
Edward Stites
Anna Stöckl
Thomas Stockner
Steven Stoddard
Gautier Stoll
Mark Stopfer
Brad Story
Roland Stote
Ronny Straube
Thomas Stricker
Christopher Stroupe
Martin Strube-Bloss
David Strutt
Michael Stubbington
John Stufken
Michael Stumpf
Yannis Stylianou
Andrew Su
Peter Sudmant
Piotr Suffczynski
Cassidy Sugimoto
Christopher Summerfield
Ren Sun
Kaustubh Supekar
Richard Superfine
Ivan Surovtsev

Christopher Surratt
Neil Swainston
Yann Sweeney
David Swigon
Eörs Szathmáry
Ewa Szczurek
Camille Szmaragd
Gergely J. Szollosi
Ilias Tagkopoulos
John Tainer
Shoji Takada
Daniel Takahashi
Atsuko Takamatsu
Ken Takiyama
Kohtaro Tanaka
Yinjie Tang
Yun Tao
Peng Tao
Sonia Tarazona
Corina Tarnita
Masami Tatsuno
Margaret Taub
Jiannis Taxidis
Yvonne Tay
James Taylor
Jordan Taylor
Tatjana Tchumatchenko
Vladimir Teif
Amalio Telenti
Pieter Rein ten Wolde
Xiangbin Teng
Santani Teng
Brigitte Tenhumberg
Augusto Tentori
Burcu Tepekule
Tomoki Terada
David Terman
Christian Tetzlaff
Shivendra Tewari
Johannes Textor
Mukund Thattai
Marcus Thelen
Evangelos Theodorou
Jean-Philippe Thivierge
David Thomas
Peter Thomas
S. Randall Thomas
Barry Thompson
Toby Thorne
Thomas Thorne
Tianhai Tian
Guido Tiana
Bruce Tidor
Kathryn Tiedje
D. Peter Tieleman
Irene Tiemann-Boege
Paul Tiesinga
Mikhail Tikhonov
Nicholas Timme
Jens Timmer
Yulia Timofeeva
Michele Tizzoni
Philippe Tobler
Nobuhiko Tokuriki
Francesco Tombola
Andrija Tomovic
Alessia Tonelli
Sid Topiol
Vincent Torre
Joaquin Torres
Gelsy Torres-Oviedo
Zoë Tosi
Maxim Totrov
Emma Towlson
Mary Towner
Taro Toyoizumi
Maria Tozzi
Ngoc Tran
Leon-Charles Tranchevent
Rui Travasso
Mikael Trellet
Matthew Tresch
Alessandro Treves
Shailesh Tripathi
Brett Trost
Wilson Truccolo
Krasimira Tsaneva-Atanasova
Konstantinos Tsetsos

Nikolaos M. Tsoukias
Mark Tuckerman
Tamir Tuller
Jerold Turner
Jack Tuszynski
Jason Tylianakis
Ghanim Ullah
Jakob Ulmschneider
David Umulis
Karan Uppal
Séverine Urdy
Sagar Utturkar
Vladimir Uversky
Nagarajan Vaidehi
Sandor Vajda
Giorgio Valentini
Catalina Vallejos
Angelo Valleriani
Sergi Valverde
Minus van Baalen
Ronald van den Berg
Frank van den Bosch
Ruud van den Brink
Martijn van den Heuvel
H. W. P. (Henk) van den Toorn
Gary Van Domselaar
Hans Van Dongen
Stephan Van Gils
Johan van Hasselt
Jeroen van Houdt
Marc Van Hulle
Arjen van Ooyen
Bas van Opheusden
Willem Van Panhuis
Jaap van Pelt
Michel van Putten
Mark van Rossum
Herman van Vlijmen
Virginie van Wassenhove
Jeroen Van Zon
John Vandermeer
Carlo Vanderzande
Fabio Vandin
Rufin VanRullen
Brian Varisco
Pablo Varona
Nelle Varoquaux
Gael Varoquaux
Rohit Vashisht
Ilpo Vattulainen
Marc Vaudel
Timothy Vaughan
Dimitrios Vavylonis
Alexei Vazquez
Jorge Velasco-Hernandez
Alan Veliz Cuba
K. V. Venkatesh
Massimo Vergassola
Elisabeta Vergu
Sergio Verjovski-Almeida
Paul Verschure
Karin Verspoor
Petra Vertes
Alessandro Vespignani
Christian Vestergaard
Martina Vettoretti
Andrew Vickers
Jonathan D. Victor
Mauno Vihinen
Jose Vilar
Andrej Vilfan
Alejandro Villaverde
Bruno Villoutreix
Martin Vinck
Martin Vingron
Sparisoma Viridi
Yoram Vodovotz
Tim Vogels
Joshua Vogelstein
Irina Voineagu
Tobias von der Haar
Max von Kleist
Jiri Vondrasek
Anja Voss-Böhme
Gregory Voth
Jocelyne Vreede
Gert Vriend
Renske Vroomans

Josef Wachtveitl
Wytse Wadman
Daniel Wagenaar
Sam Walcott
Edgar Walker
Mark Wallace
Jacco Wallinga
Dirk Walther
Wenqi Wang
Zichen Wang
Kanix Wang
Yijie Wang
Xuefeng Wang
Xiao Wang
Jin Wang
Wen Wang
Wenyi Wang
Fan Wang
Chun-Chao Wang
Enzo Wanke
Aryeh Warmflash
Catherine Warnaby
Mark Wass
Geoffrey Wasteneys
Takamitsu Watanabe
Alice Wattam
Steven Watterson
Richard Waugh
David Waxman
Ben Webb
Frederik Weber
Kyle Wedgwood
Suzanne Weekes
Kunlin Wei
Wei Wei
Martin Weigt
Aaron Weimann
Leor Weinberger
Daniel Weinberger
Daniel Weinreich
Jonathan Weinstein
Harel Weinstein
Lena Welsh
Paul Werginz
Steffen Werner
Benjamin Werner
Amy Wesolowski
Ralf Wessel
John Westbrook
Kirstie Whitaker
Stephen White
Timothy Whitehead
John Whitney
Michael Wibral
Felix Wichmann
Martin Wiener
Siouxsie Wiles
Steven Wiley
Lander Willem
Amy Williams
Jason Williams
Ben Willmore
Peter Wills
Corey Wilson
Greg Wilson
Robert Wilson
William Wilson
G. Elliott Wimmer
Elliott Wimmer
Klaus Wimmer
Alan Wing
David Winogradoff
Raimond Winslow
Robert Wiseman
Shoshana Wodak
Dominik Wodarz
Florentin Woergoetter
Jana Wolf
Charles Wolgemuth
Olaf Wolkenhauer
Roy Wollman
Thilo Womelsdorf
Kyoung-Jae Won
Chung Wong
Lee-Jun Wong
Jerome Wong-Ng
Kevin Wood
Thomas Woolley

Jonathan Wren
Yilin Wu
Sheng Wu
Di Wu
Si Wu
Baojun Wu
Anton Wutz
Ryan Wylie
Joao Xavier
Ruibin Xi
Jianguo Xia
Yu Xia
Kehui Xiang
Feifei Xiao
Zhengwei Xie
Jun Xu
Yangwen Xu
Jinbo Xu
Qifang Xu
Katherine Xue
Oded Yaakobi
Laith Yakob
Lev Yampolsky
Pengyi Yang
Sichun Yang
Guangyu Yang
Zhenbiao Yang
Laurence Yang
Jialiang Yang
Qiong Yang
Chen Yanover
Guang Yao
Yosi Yarom
Christian Yates
Andrew Yates
Todd Yeates
Laising Yen
Pierre Yger
Tau-Mu Yi
Jian Yin
Nir Yosef
Edgar Young
Alistair Young
Yuan-Nan Young
Jiyang Yu
Lianbo Yu
Byron Yu
Alan Yu
Guoxian Yu
Guo-Cheng Yuan
Can A. Yucesoy
Feng Yue
Christina Yung
Elizabeth Yuriev
Vasily Zaburdaev
Margarita Zachariou
Bojan Zagrovic
Muhammad Zaman
Johannes Zanker
Stefano Zapperi
Mihaela Zavolan
Jonathan Zelner
Chen Zeng
Karsten Zengler
Friedemann Zenke
Mingjie Zhang
Qi Zhang
Chi Zhang
Peng Zhang
Hang Zhang
Lei Zhang
Ying Zhang
Bin Zhang
Fangyuan Zhang
Bing Zhang
Henggui Zhang
Zhaolei Zhang
Byoung-Tak Zhang
Shihua Zhang
Tongli Zhang
Qingpeng Zhang
Changwang Zhang
Qi Zhao
Guiping Zhao
Li Zhaoping
Fan Zheng
Wenjun Zheng
Alexander Zhigalov

Yi Zhong
Guanglu Zhou
Xiang Zhou
Xiaobo Zhou
Changsong Zhou
Lei Zhou
Yaoqi Zhou
Qiang Zhu
Daniel Zielinski
Anton Zilman
Ralf Zimmer
Christophe Zimmer
Naupaka Zimmerman
Giuseppe Zito
Michal Zochowski
Ali Zomorrodi
Simona Zompi
Joel Zylerberberg
